# Healthcare providers’ confidence, satisfaction, and perceived preparedness following a multicomponent simulation-based cardiopulmonary resuscitation training program in Gaza

**DOI:** 10.3389/fpubh.2026.1950292

**Published:** 2026-09-29

**Authors:** Madleen Jawad Sobhe Abu Aser, Sharif Jamal Salha, Hasan Husam Ibrahim Al-Balawi

**Affiliations:** 1 Islamic University of Gaza, Gaza, Palestine; 2 Al-Esraa University, Gaza, Palestine; 3 Al-Quds University, Jerusalem, Palestine; 4 Medical Aid for Palestinians (MAP-UK), London, United Kingdom

**Keywords:** cardiopulmonary resuscitation, confidence, emergency medicine, Gaza, healthcare providers, preparedness, satisfaction, simulation-based education

## Abstract

**Background:**

High-quality cardiopulmonary resuscitation (CPR) requires not only technical competence but also confidence, effective teamwork, and psychological readiness among healthcare providers. Evidence regarding the impact of simulation-based CPR training on healthcare providers’ confidence, satisfaction, and perceived preparedness remains limited in conflict-affected and resource-constrained settings.

**Objective:**

To assess healthcare providers’ self-confidence, satisfaction, and perceived preparedness following an intensive, multicomponent simulation-based CPR training program implemented among physicians and nurses working in governmental critical care facilities in Gaza.

**Methods:**

This was a single-group evaluation of an intensive, 8-h multicomponent simulation-based CPR training program delivered to 100 healthcare providers (50 physicians, 50 nurses) working in emergency rooms, intensive care units, and coronary care units at Al-Shifa Medical Complex, Gaza, between August and December 2022, aligned with the 2020 American Heart Association Guidelines. Participants were recruited using purposive, non-probability sampling of all eligible clinical staff in these units. Self-reported confidence in CPR-related competencies was assessed before and after the intervention using identical questionnaire items; satisfaction and perceived preparedness were assessed only after the intervention. Outcomes were measured on a structured, five-point Likert-scale questionnaire (Cronbach’s *α* = 0.87), content-validated by an expert panel and refined through a pilot study, analyzed descriptively using SPSS version 26.

**Results:**

Participants reported very high satisfaction across all training domains, with instructor feedback usefulness rated highest (4.75 ± 0.39). Self-reported confidence increased markedly from pre- to post-training across all assessed domains: confidence in performing CPR rose from 2.91 ± 0.82 to 4.63 ± 0.51, airway management confidence from 2.85 ± 0.79 to 4.58 ± 0.54, and CPR team leadership confidence from 2.73 ± 0.88 to 4.49 ± 0.57. Most participants (92%) preferred simulation-based training over traditional lecture-based methods, and post-training perceived preparedness for real cardiac arrest situations was high (rapid response ability, 4.71 ± 0.40).

**Conclusion:**

Multicomponent simulation-based CPR training was associated with marked improvement in healthcare providers’ self-reported confidence, and was accompanied by high levels of post-training satisfaction and perceived emergency preparedness in a conflict-affected healthcare environment. Integrating structured simulation-based education into continuing professional development programs may strengthen emergency response capacity and workforce readiness in resource-limited settings.

## 1 Introduction

Cardiac arrest remains one of the leading causes of preventable mortality worldwide, and survival depends heavily on the rapid initiation of high-quality cardiopulmonary resuscitation (CPR). Effective CPR requires healthcare providers to possess adequate technical skills, clinical decision-making abilities, and the capacity to function efficiently within multidisciplinary resuscitation teams. The American Heart Association emphasizes that early recognition, immediate chest compressions, rapid defibrillation, effective ventilation, and coordinated teamwork are essential components of successful resuscitation systems (1).

Despite widespread availability of CPR training programs, maintaining long-term competency among healthcare providers remains challenging. Studies have demonstrated that CPR performance quality may deteriorate over time, even among trained professionals, highlighting the need for continuous education and repeated skills reinforcement (2–4). International consensus recommendations emphasize that resuscitation education should move beyond traditional knowledge-based approaches toward competency-focused strategies incorporating deliberate practice, feedback, teamwork training, and simulation-based learning (5, 6).

Traditional healthcare education has frequently relied on lectures and passive knowledge transfer. Although these approaches may improve theoretical understanding, they provide limited opportunities for healthcare providers to practice complex emergency decision-making and teamwork behaviors. Simulation-based medical education addresses these limitations by creating realistic clinical environments where participants can repeatedly practice technical and non-technical skills, receive immediate feedback, and reflect through structured debriefing (5, 7).

The importance of simulation-based education becomes even greater in conflict-affected and resource-constrained healthcare systems. Healthcare providers working in humanitarian settings often face limited access to continuing professional development, workforce shortages, restricted training opportunities, and repeated exposure to high-acuity emergencies (8). The healthcare system in Gaza represents a particularly challenging environment characterized by recurrent emergencies, limited resources, and substantial pressure on frontline medical teams. Previous research conducted in Gaza demonstrated the effectiveness of simulation-based Basic Life Support (BLS) training among nurses, showing significant improvements in knowledge following structured educational interventions (9). However, previous research has primarily focused on knowledge acquisition among nursing populations, while important affective outcomes—including confidence, satisfaction, and perceived preparedness—remain insufficiently explored across both physicians and nurses.

The present study addresses these knowledge gaps through an evaluation of a multicomponent simulation-based CPR training program among a multidisciplinary group of physicians and nurses working in emergency and critical care settings in Gaza, focusing specifically on healthcare providers’ confidence, satisfaction, and perceived preparedness following training.

### 1.1 Study aim

This study aimed to evaluate healthcare providers’ confidence, satisfaction, and perceived preparedness following a multicomponent simulation-based CPR training program among physicians and nurses working in governmental critical care facilities in Gaza.

### 1.2 Hypothesis

Multicomponent simulation-based CPR training improves healthcare providers’ self-confidence and is associated with high satisfaction and perceived preparedness for emergency cardiac arrest management.

## 2 Methods

### 2.1 Study design

This study was conducted as a single-group evaluation assessing self-reported outcomes following a multicomponent simulation-based cardiopulmonary resuscitation (CPR) training program among healthcare providers in Gaza. All enrolled participants received the same training intervention; no separate control or comparison group was included. Self-reported confidence was assessed immediately before and immediately after the intervention using identical questionnaire items; satisfaction and perceived preparedness were assessed only after completion of the intervention.

### 2.2 Study setting

The study was conducted in governmental hospitals in the Gaza Strip, Palestine, among staff of the Ministry of Health (MOH). Participants were recruited from high-acuity clinical departments responsible for emergency stabilization and resuscitation care, including Emergency Rooms (ERs), Intensive Care Units (ICUs), and Coronary Care Units (CCUs). The study was conducted at Al-Shifa Medical Complex, the largest tertiary referral hospital in Gaza and a major provider of emergency and critical care services. Recruitment, training, and data collection took place between 1 August 2022 and 10 December 2022.

### 2.3 Participants

A total of 100 healthcare providers participated in the study, including physicians (*n* = 50, 50%) and nurses (*n* = 50, 50%) directly involved in emergency and critical care services. Participants were identified using a purposive, non-probability sampling approach: all doctors and nurses employed in the ER, ICU, and CCU of the participating MOH governmental hospital who met the eligibility criteria were invited to participate. The equal physician–nurse split reflects the composition of eligible staff invited from these departments rather than a fixed quota. Full demographic and professional-background characteristics are summarized in Table 1 and Figure 1.

**Table 1:** Baseline demographic and professional characteristics of participants (*n* = 100).

Variable	Category	*n* (%)
Sex	Male	78 (78%)
	Female	22 (22%)
Age	Under 30 years	66 (66%)
	30 years and above	34 (34%)
Profession	Physicians	50 (50%)
	Nurses	50 (50%)
Education level	Bachelor’s degree	75 (75%)
	Other/higher degree	25 (25%)
Clinical department	Emergency Room (ER)	40 (40%)
	Intensive Care Unit (ICU)	30 (30%)
	Coronary Care Unit (CCU)	30 (30%)
Years of professional experience	1–5 years	52 (52%)
	6–9 years	28 (28%)
	More than 10 years	20 (20%)
Holds a CPR certificate	Yes	87 (87%)
	No	13 (13%)
Completed a dedicated CPR course (of those certified, *n* = 87)	Yes	74 (85.1%)
	No	13 (14.9%)
Time since most recent BLS/ILS/ALS course	Less than 1 year	51 (51%)
	1–2 years	31 (31%)
	More than 2 years	18 (18%)

**Figure 1:**
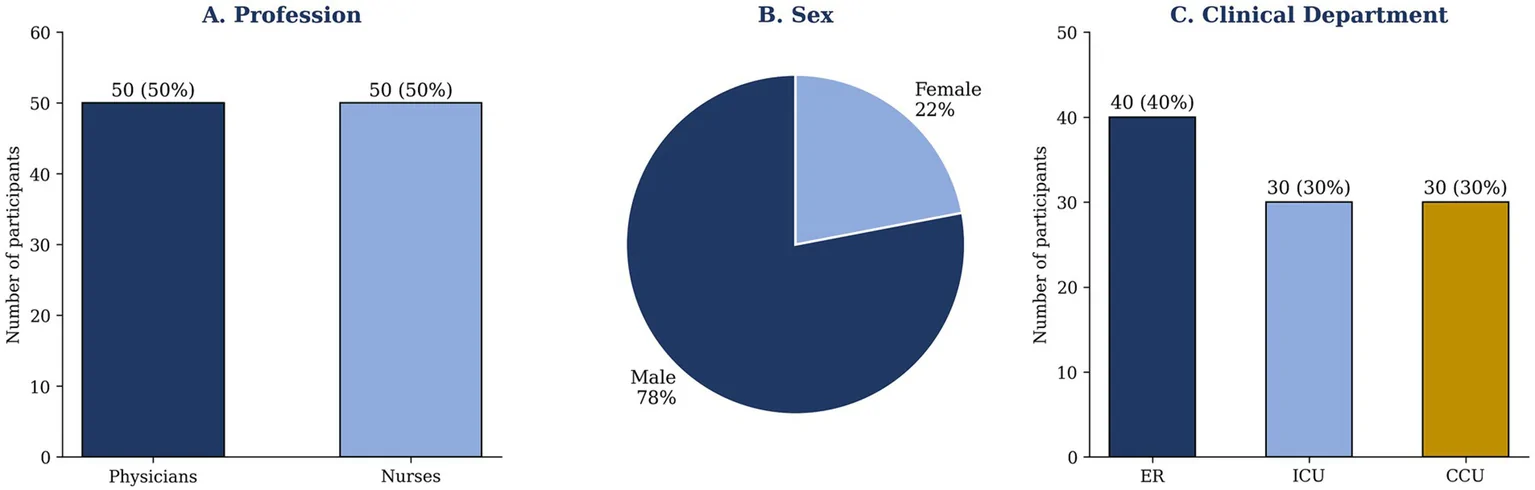
XXX.

*Inclusion criteria*: active clinical practice in the ER, ICU, or CCU of a participating MOH governmental hospital in Gaza, and willingness to participate.

*Exclusion criteria*: trainees or volunteer staff, and staff employed for less than one year.

### 2.4 Intervention

Participants completed an intensive 8-h multicomponent simulation-based CPR training program, delivered in a single day at the hospital’s simulation training center, developed according to the (1) Guidelines for Cardiopulmonary Resuscitation and Emergency Cardiovascular Care. The program was delivered by a single instructor (the lead investigator), a certified trainer across multiple Basic and Advanced Life Support programs, and consisted of:

- Structured theoretical instruction: updated CPR concepts, guideline-based recommendations, and principles of cardiac arrest management, drawn from 2020 AHA BLS and ACLS course content.
- Video-based demonstrations reinforcing correct CPR technique and emergency response procedures.
- Hands-on CPR and AED training using CPR training manikins: chest compression technique (the CAB approach), ventilation and basic airway management, and automated external defibrillator (AED) use, adapted directly from AHA BLS course content.
- Team-based simulation scenarios: realistic cardiac arrest scenarios requiring team communication, leadership, clinical decision-making, and role allocation during resuscitation.
- Structured debriefing: facilitator-guided sessions after simulation exercises to encourage reflection, identify performance gaps, and improve clinical reasoning.

Training standardization was maintained through predefined scenarios, an instructor checklist, and structured educational materials (Figure 2).

**Figure 2:**
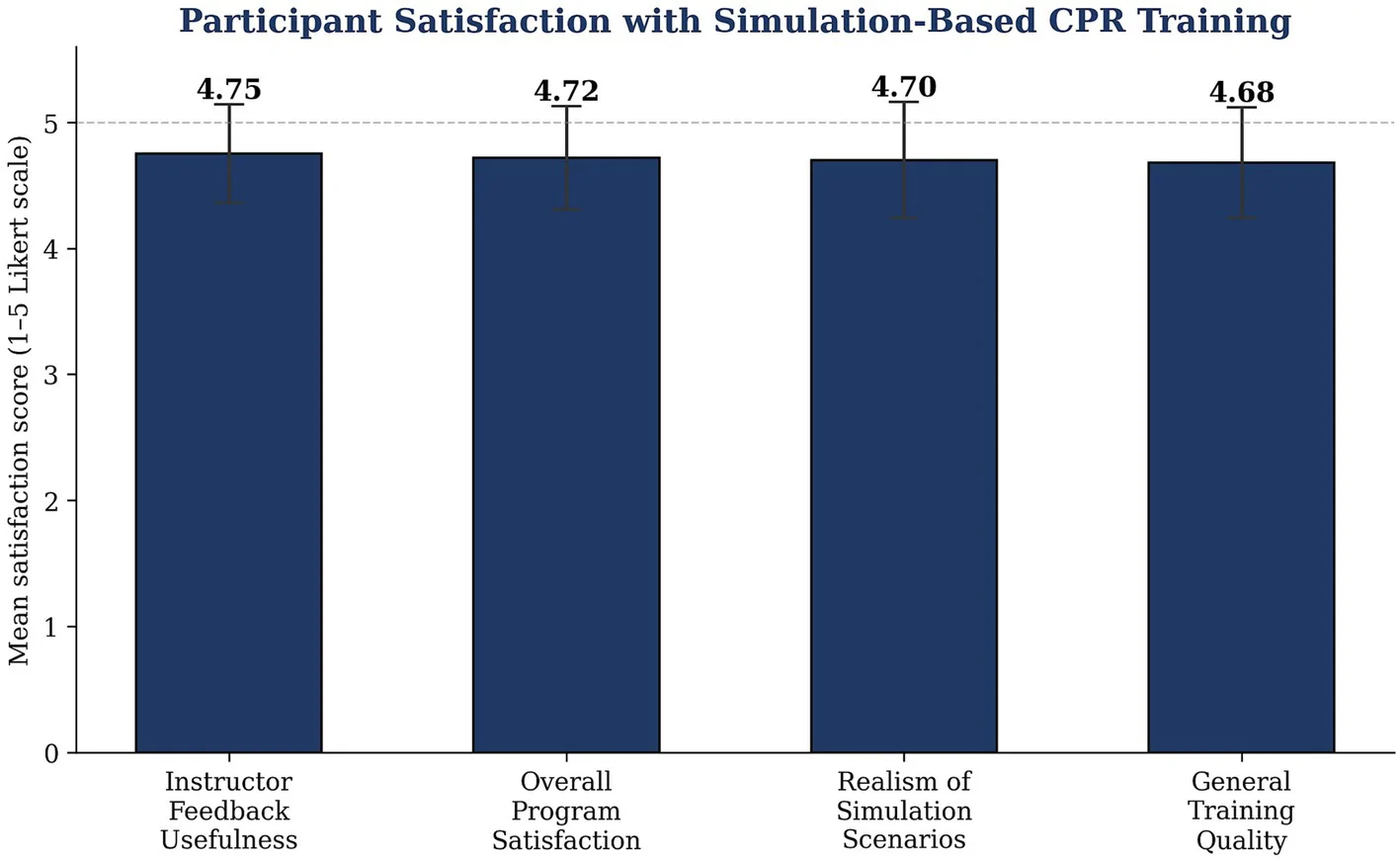
Participants’ satisfaction scores across evaluated training domains.

### 2.5 Outcome measures

Primary outcomes were self-reported confidence in CPR-related competencies and satisfaction with the training. Secondary outcomes were perceived preparedness for real cardiac arrest situations and preference for simulation-based versus traditional educational approaches. Confidence in CPR-related competencies (ability to perform CPR, airway management, and team leadership) was assessed using identical questionnaire items administered immediately before and immediately after the intervention. Satisfaction with the training and perceived preparedness for real cardiac arrest situations were assessed only after completion of the intervention. All items were rated on a five-point Likert scale.

Questionnaire items were developed by the study team based on the 2020 AHA guidelines and the training curriculum. Content validity was reviewed by a panel of 10 specialists from the Islamic University of Gaza and the Ministry of Health, and items were revised according to their feedback. A pilot study involving 10% of the target sample was conducted beforehand to assess clarity and feasibility; pilot responses were excluded from the main analysis. Test–retest reliability was additionally assessed among a subset of participants. The internal consistency of the overall instrument was high (Cronbach’s *α* = 0.87); reliability was not calculated separately for each domain (confidence, satisfaction, preparedness), which is noted as a limitation.

### 2.6 Data analysis

Data analysis was performed using SPSS version 26. Descriptive statistical methods were applied, including means, standard deviations, frequencies, and percentages. Individual pre- and post-training confidence responses were not linked by a participant identifier; paired inferential statistical testing was therefore not performed, and pre−/post-training differences are reported as observed changes in group means rather than statistically tested effects. Because this was a single-group design without a comparison group, between-group inferential comparisons were also not performed.

### 2.7 Ethical considerations

Ethical approval was obtained from the Palestinian Health Research Council Helsinki Committee (Approval number: PHRC/HC/1031/22). Institutional permission was obtained from the participating hospital and the Ministry of Health’s general directorate of human resources development. All participants provided written informed consent before enrolment and retained the right to decline or withdraw at any time. The study was conducted according to the principles of the Declaration of Helsinki.

## 3 Results

### 3.1 Training satisfaction

Participants reported high levels of satisfaction with the multicomponent simulation-based CPR training program across the evaluated domains (Table 2). The usefulness of instructor feedback received the highest mean satisfaction score (4.75 ± 0.39), followed by overall satisfaction with the training program (4.72 ± 0.41). Participants also rated the physical realism of the simulation scenarios highly (4.70 ± 0.46). Overall, these findings indicate a consistently favorable evaluation of the training experience.

**Table 2:** Participants’ satisfaction with the simulation-based CPR training program (*n* = 100).

Satisfaction domain	Mean ± SD
Usefulness of instructor feedback	4.75 ± 0.39
Overall satisfaction with the training program	4.72 ± 0.41
Physical realism of simulation scenarios	4.70 ± 0.46

Satisfaction was assessed using a five-point Likert scale, with higher scores indicating greater satisfaction.

### 3.2 Pre- and post-training self-confidence

Self-reported confidence increased across all three assessed CPR-related competency domains following completion of the simulation-based training program (Table 3; Figure 3).

**Table 3:** Self-reported confidence before and after simulation-based CPR training (*n* = 100).

Clinical competency domain	Pre-training Mean ± SD	Post-training Mean ± SD	Observed change
Ability to perform CPR	2.91 ± 0.82	4.63 ± 0.51	+1.72
Airway management confidence	2.85 ± 0.79	4.58 ± 0.54	+1.73
Team leadership during CPR	2.73 ± 0.88	4.49 ± 0.57	+1.76

Scores were based on a five-point Likert scale. The observed change represents the difference between post-training and pre-training group means; no paired statistical test was performed because individual responses could not be linked.

**Figure 3:**
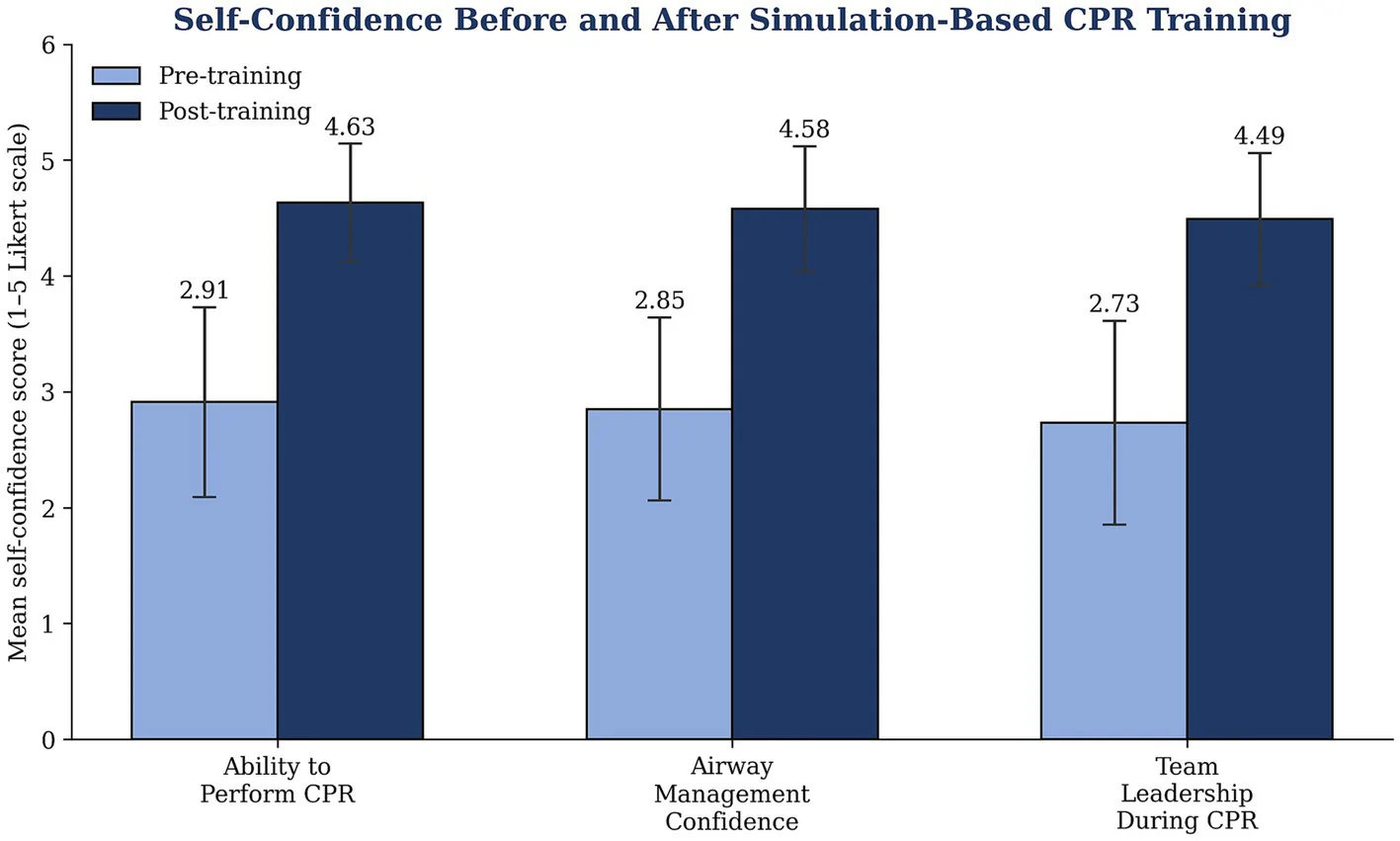
Pre- and post-training self-reported confidence scores across CPR competency domains.

Confidence in the ability to perform CPR increased from a pre-training mean score of 2.91 ± 0.82 to a post-training mean score of 4.63 ± 0.51. Similarly, confidence in airway management increased from 2.85 ± 0.79 before training to 4.58 ± 0.54 after training. The largest numerical increase was observed in confidence in team leadership during CPR, which increased from 2.73 ± 0.88 to 4.49 ± 0.57.

Because individual pre- and post-training responses were not linked using participant identifiers, paired inferential statistical testing could not be performed. Therefore, the findings are presented as observed changes in group mean scores and should not be interpreted as statistically tested intervention effects (Figure 4).

**Figure 4:**
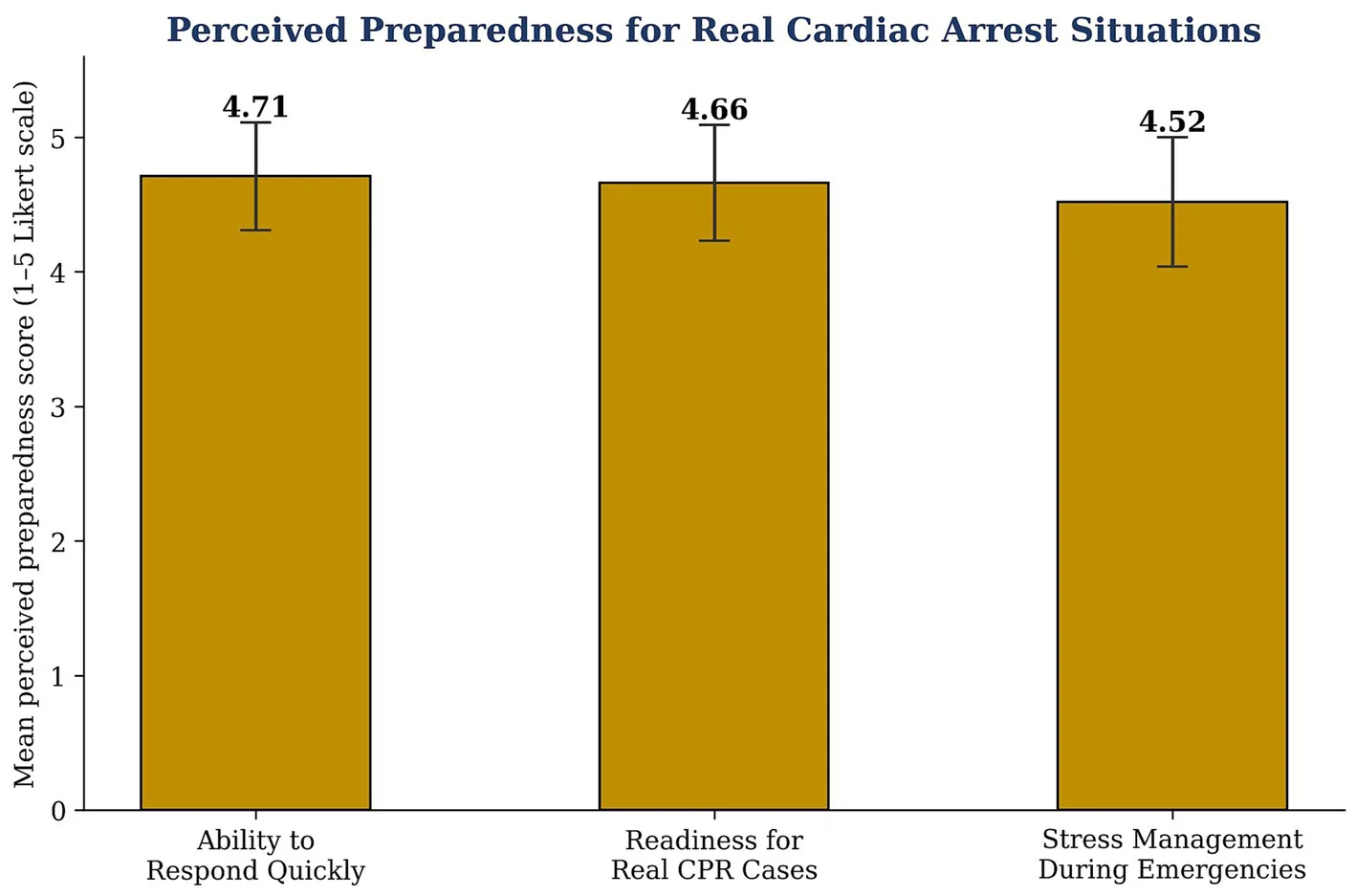
Perceived preparedness for real cardiac arrest situations following training.

### 3.3 Perceived preparedness for real cardiac arrest situations

Participants also reported high levels of perceived preparedness for managing real cardiac arrest situations following completion of the training program (Table 4). The mean score for the ability to respond rapidly to a cardiac arrest situation was 4.71 ± 0.40 on the five-point Likert scale, indicating a high level of perceived emergency readiness.

**Table 4:** Perceived preparedness for real cardiac arrest situations following simulation-based CPR training (*n* = 100).

Preparedness domain	Mean ± SD
Ability to respond rapidly to a cardiac arrest situation	4.71 ± 0.40

Preparedness was assessed after completion of the training using a five-point Likert scale, with higher scores indicating greater perceived preparedness.

### 3.4 Preferred educational modality

Participants were asked to indicate their preferred approach for future professional development. The majority (92%) preferred simulation-based training modules, whereas 8% preferred traditional didactic lectures (Table 5; Figure 5). Thus, interactive simulation-based education was substantially more frequently preferred than conventional lecture-based education.

**Table 5:** Preferred professional training modality among participants (*n* = 100).

Preferred training modality	*n*	%
Simulation-based training modules	92	92%
Traditional didactic lectures	8	8%
Total	100	100%

**Figure 5:**
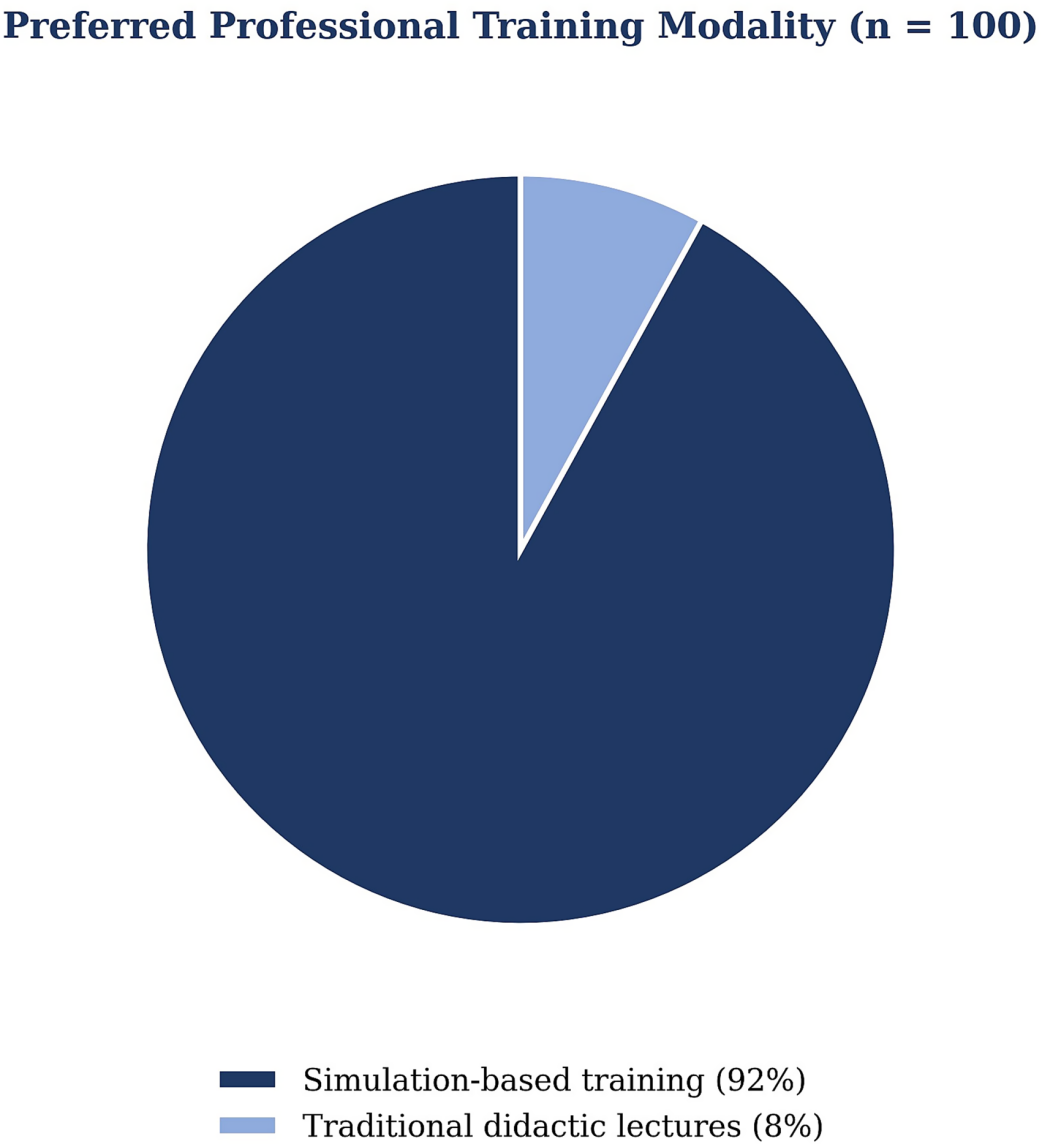
Participants’ preferred professional training modality.

### 3.5 Summary of Main findings

Overall, participants reported high satisfaction with the multicomponent simulation-based CPR training program, particularly regarding the usefulness of instructor feedback and the realism of the simulation scenarios. Self-reported confidence increased numerically across all three assessed competency domains, including CPR performance, airway management, and team leadership. Participants also reported high perceived preparedness for real cardiac arrest situations, with rapid response ability receiving a mean score of 4.71 ± 0.40. In addition, 92% of participants preferred simulation-based training over traditional didactic lectures for future professional development.

Importantly, the pre- and post-training confidence findings represent observed differences in group means rather than statistically tested paired changes because individual questionnaire responses could not be linked across the two assessment points.

## 4 Discussion

This evaluation demonstrates that a multicomponent simulation-based cardiopulmonary resuscitation (CPR) training program was associated with marked improvement in healthcare providers’ self-reported confidence and was accompanied by high satisfaction and perceived preparedness in a conflict-affected healthcare environment. The findings provide evidence that simulation-based education can strengthen self-reported confidence and psychological readiness among frontline physicians and nurses working in emergency and critical care settings. This study did not include an objective assessment of technical CPR performance—such as compression depth or rate, ventilation quality, time-to-defibrillation, or skills-checklist scores—so conclusions about technical competence cannot be drawn from these data; prior literature reporting objective psychomotor gains from simulation training (5, 7, 10) is discussed below for context only, not as a finding of the present study.

Healthcare provider confidence is an essential component of effective resuscitation performance. During cardiac arrest events, providers must rapidly integrate technical skills, clinical judgment, communication, and teamwork under significant psychological pressure. Previous research has demonstrated that CPR quality may be inadequate even among trained healthcare professionals, emphasizing the importance of continuous competency development and realistic practice environments (2–4).

The high satisfaction scores observed in this study support previous evidence that simulation-based learning is well accepted by healthcare professionals. Participants particularly valued instructor feedback and scenario realism, suggesting that experiential learning environments may offer benefits beyond traditional knowledge-based approaches (5, 7).

The increase in self-reported confidence across CPR performance, airway management, and team leadership domains is consistent with international evidence supporting simulation-based resuscitation education, including recommendations from Cheng et al. (5) and the International Liaison Committee on Resuscitation (6) favoring competency-based, simulation-supported learning over passive information delivery.

The demographic and professional-background characteristics of participants reflect the healthcare workforce involved in emergency care delivery in Gaza: the majority had 1–5 years of clinical experience (52%), most held a CPR certificate (87%), and just over half had completed a BLS/ILS/ALS refresher within the past year (51%), suggesting the cohort was broadly familiar with CPR concepts before the intervention, against which the observed gains in self-reported confidence can be interpreted.

The current findings are consistent with previous research conducted in Gaza. Fahajan et al. (9) demonstrated significant improvement in knowledge following simulation-based Basic Life Support training among nurses working in governmental hospitals in the Gaza Strip. The present study expands on this evidence by evaluating both physicians and nurses and focusing on confidence, satisfaction, and perceived preparedness.

The preference of 92% of participants for simulation-based training over traditional lectures highlights the perceived educational value of immersive learning approaches, particularly relevant in resource-constrained settings where continuing professional development opportunities may be limited (8).

Implementation of simulation-based CPR training in humanitarian settings requires sustainable institutional commitment, availability of trained instructors, regular refresher programs, and integration into formal continuing professional development systems. Although participants demonstrated substantial improvements in self-reported confidence, the long-term sustainability of these gains was not assessed in this study. Previous research has shown that CPR and BLS skills may decline over time without reinforcement, supporting recommendations for repeated refresher training and continuous competency assessment (1, 11, 12).

## 5 Strengths and limitations

A strength of this study is its inclusion of both physicians and nurses from multiple critical care settings (ER, ICU, and CCU), enhancing the applicability of the findings across multidisciplinary healthcare teams. The use of a structured, content-validated, and pilot-tested instrument with high overall internal consistency (Cronbach’s *α* = 0.87) also strengthens confidence in the outcome measures.

Several limitations should be acknowledged. First, this was a single-group design without a control or comparison group, and pre- and post-training confidence responses could not be linked at the individual level, so paired inferential statistical testing was not possible; observed improvements cannot be definitively attributed to the training intervention alone, as factors such as testing effects or natural growth in confidence over time cannot be ruled out. Second, the study was conducted in a single tertiary hospital within one geographic region, which may limit generalizability. Third, long-term retention of confidence and skills was not evaluated. Fourth, clinical patient outcomes such as return of spontaneous circulation (ROSC) and survival rates were not measured. Fifth, this study did not include an objective assessment of CPR technical performance. Sixth, the outcome questionnaire, while content-validated, pilot-tested, and internally consistent overall, did not have reliability calculated separately for each domain. Future studies should incorporate a comparison group, individually linked pre−/post-training data with formal paired inferential testing, longitudinal follow-up, and evaluation of the intervention’s impact on objective technical performance and patient outcomes.

## 6 Conclusion and recommendations

Multicomponent simulation-based CPR training was associated with marked improvement in the self-reported confidence of healthcare providers practicing in Gaza’s public hospitals, and was accompanied by high satisfaction and perceived emergency readiness. Moving toward immersive, hands-on learning may help clinicians manage both the technical demands and the psychological stress of real-life resuscitation events.

Based on these findings, the following recommendations are proposed:

- Mandatory policy integration: shift continuing education requirements within governmental hospitals away from passive lectures, making simulation-based CPR training a standard certification component.
- Regular refresher tracks: implement short, mandatory simulation refresher sessions approximately every 6 months to counter skill decay and maintain confidence.
- Broaden institutional reach: expand simulation equipment and training programs beyond central hubs into regional emergency, trauma, and secondary care departments across the territory.
- Long-term follow-up research: conduct future prospective, controlled studies with individually linked pre−/post-training data, formal paired statistical testing, and objective performance assessment, to track how gains in confidence and readiness are sustained over time and relate to patient outcomes.

## Data Availability

The datasets presented in this study can be found in online repositories. The names of the repository/repositories and accession number(s) can be found in the article/supplementary material.
